# Aryl Hydrocarbon Receptor Deficiency in an Exon 3 Deletion Mouse Model Promotes Hematopoietic Stem Cell Proliferation and Impacts Endosteal Niche Cells

**DOI:** 10.1155/2016/4536187

**Published:** 2016-06-16

**Authors:** Zeenath Unnisa, Kameshwar P. Singh, Ellen C. Henry, Catherine L. Donegan, John A. Bennett, Thomas A. Gasiewicz

**Affiliations:** Department of Environmental Medicine, University of Rochester Medical Center, Rochester, NY 14642, USA

## Abstract

The aryl hydrocarbon receptor (AHR) is a ligand-activated transcription factor belonging to the Per-Arnt-Sim (PAS) family of proteins. The AHR is involved in hematopoietic stem cell (HSC) functions including self-renewal, proliferation, quiescence, and differentiation. We hypothesize that AHR impacts HSC functions by influencing genes that have roles in HSC maintenance and function and that this may occur through regulation of bone marrow (BM) niche cells. We examined BM and niche cells harvested from 8-week-old AHR null-allele (KO) mice in which exon 3 was deleted in the* Ahr* gene and compared these data to cells from B6 control mice; young and old (10 months) animals were also compared. We report changes in HSCs and peripheral blood cells in mice lacking AHR. Serial transplantation assays revealed a significant increase in long term HSCs. There was a significant increase in mesenchymal stem cells constituting the endosteal BM niche. Gene expression analyses of HSCs revealed an increase in expression of genes involved in proliferation and maintenance of quiescence. Our studies infer that loss of AHR results in increased proliferation and self-renewal of long term HSCs, in part, by influencing the microenvironment in the niche regulating the balance between quiescence and proliferation in HSCs.

## 1. Introduction

All hematopoietic lineages arise from a small population of multipotent cells, the long term hematopoietic stem cells (LTHSCs) that are capable of self-renewal and differentiation. Through the process of multilineage differentiation, these HSCs develop into progenitor populations and lineage committed cells, the latter of which constitute the mature phenotype of blood and the immune system [1]. Hematopoiesis is, in part, regulated by interactions among the different cell populations constituting the bone marrow (BM) niche that balances the quiescence, proliferation, and differentiation of HSCs [2]. However, abnormal niche function can contribute to hematopoietic disease [3]. Several transcription factors mediate differentiation signals elicited by various inter- and intracellular factors and direct HSC lineage commitment. One such factor proposed to be involved in maintenance of self-renewal and proliferation of HSCs is the aryl hydrocarbon receptor (AHR) [4].

The AHR is a basic helix loop helix transcription factor belonging to the PAS (Per-Arnt-Sim) superfamily of proteins. These PAS domain proteins have an important role in circadian rhythms, organ development, neurogenesis, oxidation-reduction status, and response to hypoxia [5]. The PAS domain of AHR mediates ligand binding, eliciting translocation to the nucleus and dimerization with the AHR nuclear translocation protein (Arnt) to modulate gene transcription [6].

The AHR has been well studied for its role in mediating toxic responses to environmental xenobiotics such as 2,3,7,8-tetrachlorodibenzo-*p*-dioxin (TCDD), polycyclic aromatic hydrocarbons (PAHs), and polychlorinated biphenyls (PCBs). In order to understand the physiological function of AHR independently of xenobiotic exposure, several labs generated* Ahr* null-allele (knockout, KO) mice using different strategies [7–9]. All these mice have shown phenotypic alterations in hepatic development, reproductive health, immunology, and vascular biology compared to wild type (WT) mice. However, some differences in the degree of phenotypic change and age-dependence of these phenotypes between KO models have been observed, possibly due, at least in part, to differences in genetic background [10]. One consistent feature among these models is altered immune system function and phenotype, although, again, the specific type and degree of immune alteration may differ [11].

In previous investigations, it was observed that lack of AHR in the “Bradfield” KO mice (B6.129-*Ahr* ^*tm1Bra/J*^), in which there is a deletion of exon 2 in the* Ahr* gene [7], alters the gene expression profile of the most primitive progenitors belonging to LTHSCs [12] and also leads to altered expression of genes associated with myeloproliferative disorders in aging mice as well as shorter lifespans [13]. So far, the specific role of AHR in regulating hematopoiesis is still not completely established and is actively being investigated.

In studies described here, we examined the role of AHR in regulating hematopoiesis using recently generated AHR-KO mice that have an* Ahr* gene exon 3 deletion. Breeding strategies have determined that these mice can be generated by mating homozygote pairs, resulting in a better birth and survival rate compared to other models. Using this model we analyzed functions of HSC and compared them with previous existing models. If results in these two different strains are found to be comparable, it would strengthen the conclusion that AHR is critical in HSC function and that earlier findings are not just peculiar to the one mutant mouse (exon 2 deleted model) and also provides another strain of AHR null-allele mouse to use for studying AHR functions that might avoid the breeding problems with the exon 2 KO mice.

Based on previous investigations, we hypothesize that AHR is responsible for functional and mechanistic regulation of HSC quiescence and proliferation. We sought to determine whether immune alterations would be observed in the exon 3 deletion model and whether these would be similar to the other KO models. If so, the use of the exon 3 KO model would greatly expedite further work to understand mechanisms involved. It is known that hematopoiesis is regulated by the interactions between the different cell populations constituting the BM niche [2]. Based on this, we further hypothesized that alterations in these niche populations might be observed in KO mice. Data reported here indicate that the AHR plays an important role in HSC quiescence, proliferation, and* in vivo* HSCs BM reconstitution ability. Furthermore, deletion of a functional AHR results in gene expression changes associated with hyperproliferation, leukemia, and accelerated aging. We also report that AHR may modulate, directly or indirectly, cell populations within the BM niche.

## 2. Materials and Methods

### 2.1. Mice

All mice used for these studies were females, purchased at 6–8 weeks of age from Taconic Farms (Germantown, NY). C57BL/6-*Ahr* ^*tm1.2Arte*^ mice carry a deletion in exon 3 of the* Ahr* gene, resulting in an out of frame splicing of exons 2 to exon 4. Results were compared to* Ahr* WT mice (C57BL/6N Tac). For adoptive transfer studies, the B6.SJL-*Ptprca*/BoyAiTac (CD45.1^+^) and C57BL/6N Tac (CD45.2^+^) strains were used to permit donor and recipient hematopoietic cells to be distinguished. For aging studies, 10-month-old female mice were used. Animal handling and experimental procedures were carried out in accordance with Institutional Animal Care and Use Committee at the University of Rochester.

### 2.2. Organ Weights and Hematological Profile

Animals were euthanized in a CO_2_ chamber and organs (liver, thymus, and spleen) were harvested for wet weights. Blood was collected from the retroorbital venous plexus using capillary tubes and drained into microtainer tubes containing EDTA (Becton, Dickinson [BD] and Company, NJ). The complete blood count and other hematological parameters were analyzed using a HESKA Hematology Analyzer (HESKA Corporation, Colorado). The relative organ weights (g/100 g body weight) were calculated for the mice.

### 2.3. Bone Marrow and Spleen Cell Isolation

Bone marrow cells were harvested from femurs and tibiae by crushing the bones with a mortar and pestle. The cell suspension was collected after filtering through 40 *μ* membrane filter (BD). These cells were used for surface staining after red blood cell (RBC) lysis treatment with Red Blood Cell Lysing Buffer Hybri-Max*™* (Sigma). Spleens were crushed between two glass slides and isolated cells were used for surface staining with respective antibodies after RBC lysis. Surface staining was done with B220 (RA3-6132, BD), CD3 (145-2C11, BD), Gr-1 (RB6-8C5, BD), Mac1 (M170, BD), CD45.1 (A20, BD), and CD45.2 (104, BD). After surface staining with respective fluorescent antibodies, polychromatic flow acquisition was performed using a LSR II instrument (BD) at the University of Rochester Flow Cytometry Core. Flow data was analyzed using FlowJo software (version 10.0.7 Treestar, Ashland, OR).

### 2.4. Histology

Tissues were harvested and fixed in 10% neutral buffered formalin and processed for histology. Fixed tissues were embedded in paraffin, and 5-*μ*m sections were prepared. Sections were stained with hematoxylin and eosin for histopathological evaluation.

### 2.5. Cell Cycle Analysis

Bone marrow cells were analyzed for cell cycle phases after lineage depletion and surface staining, followed by fixation and permeabilization and staining with DAPI (4-6-diamidino-2-phenylindole, dihydrochloride, Molecular Probes). Lin^−^ cells were obtained by blocking the Fc*γ*III/II receptor using antibody clone 2.4 G2 and then incubated with biotinylated antibodies against Mac-1/CD11b (clone M1/70), B220 (RA3-6B2), Gr-1 (RB6-8C5), CD3*ε* (500A2), and Ter-119 (Ter-119) followed by streptavidin microbeads (Miltenyi Biotec). The cells were passed through magnetic columns (Miltenyi Biotec) to collect the lineage-depleted cells, and surface staining was done with fluorescently labeled stem cell antigen (Sca1) (clone D7, Biolegend) and stem cell factor (ckit) (288, BD) antibodies. The cells were cytofixed and permeabilized (BD cytofix/cytoperm) and then stained with 1 mg/mL DAPI at room temperature for thirty minutes. Cells were centrifuged and resuspended in PBS before acquisition on LSR II. Doublets were eliminated and single cells were considered for LSK (Lin^−^Sca1^+^cKit^+^) population that was analyzed for cell cycle phases based on differential staining ability.

### 2.6. *In Vivo* Cell Proliferation Assay

Mice were injected with 5-bromo-2′-deoxyuridine (BrdU) (0.1 mg/kg body weight) and BM cells were harvested 16 hours after injection and processed for lineage depletion. The Lin^−^ cells were surface stained with fluorochrome labeled antibodies for Sca-1 and ckit for 20 min on ice. The cells were washed, and the pellet was resuspended in DNAse solution and incubated for one hour at 37°C. After washing in PBS, the cells were resuspended in BD cytofix/cytoperm solution for 20 min on ice followed by intracellular staining with FITC-labeled anti-BrdU (BD Biosciences, Mountain View, CA). After a final wash with PBS, the cells were subjected to LSR II flow cytometry and the data analyzed with FlowJo software.

### 2.7. Differential Staining of BM Endosteal Niche Cells

Femurs and tibiae were harvested and crushed lightly as described above to remove hematopoietic cells. The bone fragments were washed with Hank's balanced salt solution (HBSS, Invitrogen) and 2% fetal bovine serum (FBS). The bones were crushed again in the above solution to detach the adherent cells, and after centrifugation, these were resuspended in Collagenase type I (Worthington Biochemical Corporation, Lakewood, NJ) solution made in HBSS at a concentration of 3 mg/mL [15]. Cells were incubated at 37°C for 1 hour in a shaking water bath at 100 rpm. The cell suspension was filtered through 70-*μ* filter and washed with HBSS. The cells were counted and lineage depletion was performed as described above. After lineage depletion, the cells were stained with Sca1 (V450 clone D7, BD Pharmingen), CD31 (FITC clone 390, BD Pharmingen), CD45 (PerCP clone 30-F11, BD Pharmingen), and CD51 (PE clone RMV-7, BD Pharmingen) surface markers. Single cells were gated and analyzed for endothelial cells (EC, Lin^−^CD45^−^CD31^+^), osteoblast cells (OBC, Lin^−^CD45^−^CD31^−^Sca1^−^CD51^+^), and mesenchymal stem cells (MSC, Lin^−^CD45^−^CD31^−^Sca1^+^CD51^+^).

### 2.8. Serial BM Transplantation Assay

Bone marrow cells (2 × 10^6^) from recipient CD45.1^+^ mice were combined with cells from donor CD45.2 wild type (WT) or KO Taconic mice at a ratio of 1 : 1 (2 × 10^6^ BM cells) and were injected intravenously in recipient CD45.1 irradiated (550 + 550 rads, 4 h apart) mice (8 mice/group). After 16 weeks, BM cells (1 × 10^6^) harvested from primary recipients were injected into irradiated CD45.1^+^ secondary recipient mice [16]. This procedure was repeated for a tertiary transplant. Bone marrow cells were harvested 16 weeks after transplantation and analyzed for the donor derived CD45.2^+^ cells in different lineages. This was repeated for secondary and tertiary transplant recipient animals. The donor BM was also analyzed for differential HSC populations after lineage depletion as described above and further staining with Sca1, cKit, and CD48 (clone HM48-1, BD), CD150 (clone TC15-12F12.2, Biolegend), and CD135 (clone A2F10.1, BD) to differentiate the multipotent progenitors populations MPP1 [LSK, CD135^−^, CD48^+^, CD150^−^], MPP2 [LSK, CD135^−^, CD48^+^, CD150^+^], short term HSCs (STHSC [LSK, CD135^−^, CD48^+^, CD150^−^]), and long term HSCs [LSK, CD135^−^, CD48^−^, CD150^+^] [17].

### 2.9. Reciprocal BM Transplantation

Chimeric mice were generated to determine the effect of AHR-dependent extrinsic factors on BM repopulation [18]. Briefly, the WT (CD45.1^+^), WT (CD45.2^+^), and AHR-KO (CD45.2^+^) recipient mice were lethally irradiated with 11 Gy (two equal divided doses of 5.5 Gy, 4 hours apart) and then injected intravenously with 2 million BM cells/mouse from donor WT (CD45.1^+^), WT (CD45.2^+^), or KO (CD45.2^+^) mice in the following experimental donor → recipient combinations: WT (CD45.1^+^) → WT (CD45.2^+^), WT (CD45.2^+^) → WT (CD45.1^+^), KO (CD45.2^+^) → WT (CD45.1^+^), and WT (CD45.1^+^) → KO (CD45.2^+^) (6–8 mice/group). The level of engraftment was measured in BM of recipient mice after 16 weeks of transplantation by analyzing the CD45.1^+^ and CD45.2^+^ cells by flow cytometry.

### 2.10. Colony-Forming Assays

Bone marrow cells were harvested from the femurs and tibiae. Following RBC lysis, these cells were seeded onto methylcellulose media M3434 (Stemcell Technologies, BC, Canada) according to the manufacturer's instructions. These colonies were scored after 10 days for their clonogenic ability to form differential colonies.

### 2.11. Real Time Quantitative PCR

Bone marrow Lin^−^Sca1^+^cKit^+^ (LSK) cells were sorted and collected in buffer RLT plus (Qiagen). These samples were then processed for RNA isolation and cDNA preparation at the University of Rochester Functional Genomics Core. Total RNA was isolated from sorted LSK cells using an RNeasy Mini Kit (Qiagen). RNA was preamplified and cDNA was produced using a WT-Ovation PicoSL kit (Nugen). For analysis, 10 ng of cDNA was used in qPCR reactions via a Bio-Rad CFX96 Real Time PCR instrument. The cDNA was then subjected to real time qPCR with standard TaqMan PCR primers and MasterMix (Applied Biosystems, Foster City, CA) for different genes. Expression of mRNA for each gene was normalized using the expression of 18S rRNA as a control endogenous gene. KO data were compared with WT data using the 2^−ΔΔCt^ approximation method.

### 2.12. Statistical Analysis

The results were analyzed and plotted using Graphpad Prism (Graphpad Inc., La Jolla, California). Statistical significance was determined using two-tailed Student's *t*-test. *P* values less than 0.05 were considered statistically significant (^*∗*^ *P* value < 0.05, ^*∗∗*^ *P* value < 0.01, and ^*∗∗∗*^ *P* value < 0.001 and ns = not significant).

## 3. Results

### 3.1. AHR Deletion Affects the Differential White Blood Cell Count and Organ Weights

**Figure 1:**
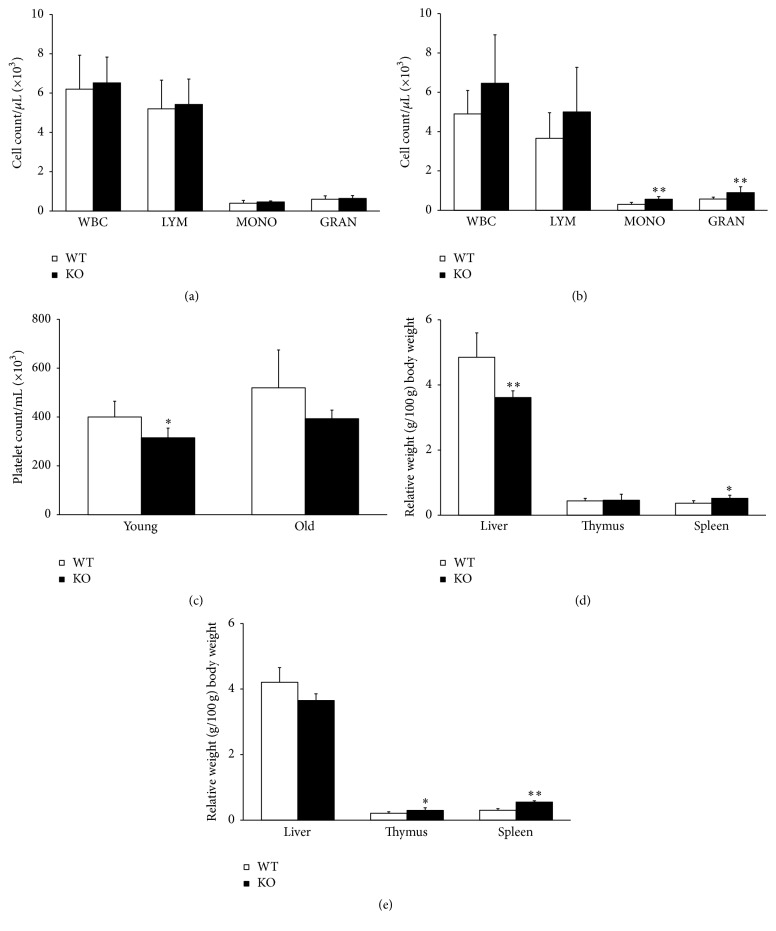
Loss of AHR expression influences the cellular content of peripheral blood and organ weights. Peripheral blood was analyzed by retroorbital bleeding and CBC differential was performed. (a) indicates cell count of young animals and (b) indicates old animals. (c) indicates differences in platelet counts. (d) represents relative weights of the hematopoietic organs and thymus in young animals. (e) represents relative weights of the hematopoietic organs and thymus in old animals. Data presented as mean ± SD, *n* = 5 mice/group. ^*∗*^Values significantly different from WT control (*P* < 0.05).

The analyses of peripheral blood for CBC differential indicated no statistical difference in the number and types of peripheral blood cells between young WT (Taconic* Ahr* wild type) and KO (Taconic* Ahr*-KO) animals (Figure 1(a)). However, there was a significant increase in the granulocytes and monocytes of aged KO animals (10 months) compared to WT age matched controls (Figure 1(b)). There was also a lower platelet count in KO animals compared to WT (Figure 1(c)), although this was statistically different only in young animals. There was a trend for decreased relative liver weights and increased thymus and spleen weights in KO mice, although this was consistently significantly different only for spleen weights in young and aged animals (Figures 1(d) and 1(e)).

### 3.2. AHR Influences HSC Proliferation and Quiescence

**Figure 2:**
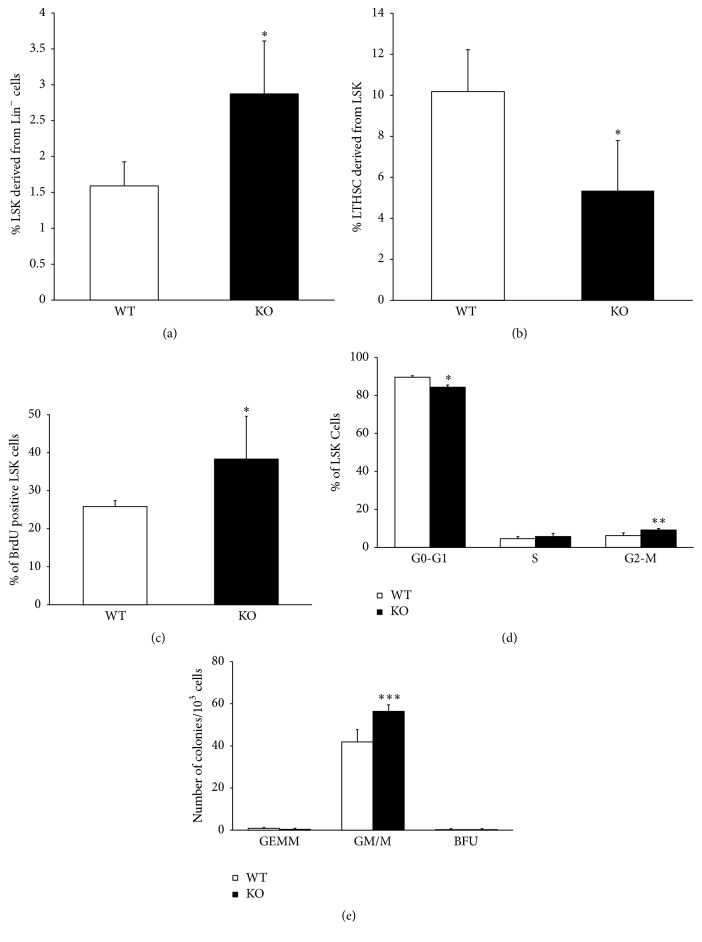
Bone marrow hematopoietic stem and progenitor cell populations, BrdU incorporation, cell cycle status, and colony-forming ability are altered by lack of AHR expression. (a) Bone marrow cells were processed for Lin^−^ cell separation, stained for specific markers, and analyzed by flow cytometry for (a) LSK and (b) LT-HSC. Bone marrow Lin^−^ cells were isolated from BrdU-treated mice, stained for LSK cells and anti-BrdU antibody, and analyzed for BrdU^+^ LSK cells (c). For cell cycle analysis, BM Lin^−^ cells were stained with DAPI and analyzed for LSK cells in G0-G1, S, and G2/M cell cycle stage (d). Colony-forming ability of primitive progenitors was assessed in BM cells (e). Data are mean ± SD, *n* = 5 mice/group. ^*∗*^Values significantly different from WT control (*P* < 0.05).

Changes in the peripheral blood may arise from alterations in the BM progenitor and/or stem populations. The BM was lineage-depleted and enumerated for the expression of Sca-1 and cKit, referred to as the progenitor- and stem cell-enriched LSK (Lin^−^Sca-1^+^cKit^+^) cells. There was an increase in the number of LSK cells in AHR-depleted animals when compared to WT (Figure 2(a)). The LSK population was further subjected to analysis for the phenotypically defined long term repopulating cells (CD135^−^CD48^−^CD150^+^) with a potential to self-renew. There was a statistically significant reduction in the phenotypic LTHSC (Figure 2(b)) in KO BM cells. But when considering the total number of LTHSC relative to LSK population it may not differ between the two groups.

Since the increased number of LSK cells could be due to increased proliferation, we determined BrdU incorporation into these cells. At 16 hours after injection, there was a significant increase in the BrdU-labeled LSK subset of cells in AHR-KO animals compared to WT (Figure 2(c)). Possible changes in LSK cell cycle were also examined. The KO animals showed a slight but significant decline in the GO/G1 phase of the cell cycle with a concomitant statistically significant increase in the G2-M phases (Figure 2(d)). These data suggest that AHR-depleted progenitor/stem cells are less quiescent and are more prone to proliferation. We also compared the clonogenic potential of whole BM cells in both groups (Figure 2(e)). In the colony-forming assay, we found no difference in the most primitive undifferentiated CFU-GEMM (granulocyte/erythrocyte/macrophage/megakaryocyte) colonies and BFU (Burst-Forming Unit-Erythroid) colonies. However, there was a significant increase in the KO group in their ability to form differentiated granulocyte/macrophage (CFU-GM/M) colonies. This suggests that the progenitor cells in KO animals have an increased ability to differentiate towards macrophages and granulocytes.

### 3.3. Functional Alteration in the AHR-KO LTHSC Population Assessed by Serial Transplantation

**Figure 3:**
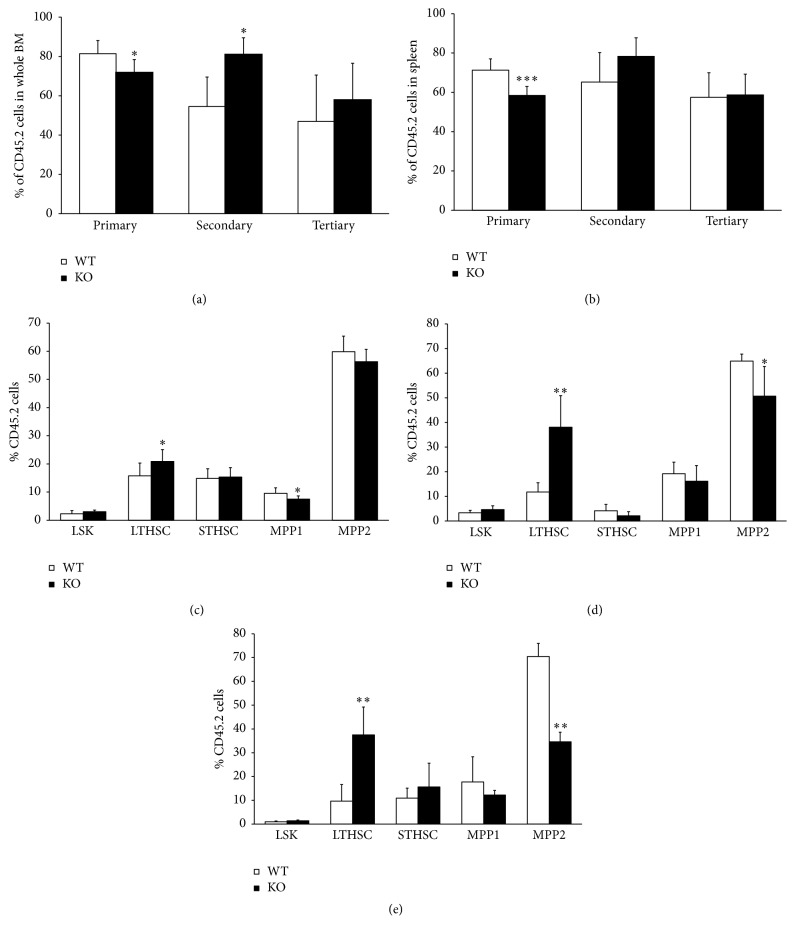
AHR-deficient BM cells have changes in long term competitive transplantation ability in serial transplantation. Bone marrow cells from donor AHR-KO (CD45.2^+^) or WT (CD45.2^+^) and recipient competitor CD45.1^+^ mice were mixed together at a ratio of 1 : 1 (1 × 10^6^ cells each) and injected into irradiated CD45.1^+^ recipient mice (8 each for donor AHR-KO and WT) for primary BM transplantation. Bone marrow cells were isolated from primary recipients after 16 weeks and serially transplanted in recipient mice as described in Section 2. (a) Bone marrow and (b) spleen cells were analyzed for CD45.2^+^ (donor) and CD45.1^+^ (recipient) origin after 16 weeks at each stage of transplantation. An analysis of BM cells was done for primitive hematopoietic and progenitor cells: (c) primary, (d) secondary bone, and (e) tertiary transplantation (LTHSC = LSK, CD135^−^, CD48^−^, CD150^+^; STHSC = LSK CD135^−^ CD48^−^ CD150^−^; MPP1 = LSK CD135^−^ CD48^+^ CD150^−^; MPP2 = LSK CD135^−^ CD48^+^ CD150^+^). Data shown are the mean ± SD. ^*∗*^Values significantly different from WT control (*P* < 0.05) (*N* = 8).

The above results indicate increased proliferation and cell cycle changes in the LSK subset. To examine the self-renewal capabilities of LTHSCs to generate more differentiated progeny, the most stringent functional test for HSCs, a serial stem cell transplantation assay was performed. The results of the primary, secondary, and tertiary transplantations analyzed 16 weeks after transplantation for donor derived cells indicate increased cell counts in the BM of secondary recipients (Figure 3(a)) and decreased spleen cell counts of the primary recipients' spleens (Figure 3(b)) of KO animals compared to WT. The cell number in tertiary transplant recipients indicated no significant difference in the cell number in both the spleen and the BM. Quantification of lineage differentiation indicated no specific lineage defects except for the slightly reduced significant B220 lineage in BM of primary KO recipients (Supplementary Figure 1D in Supplementary Material available online at http://dx.doi.org/10.1155/2016/4536187). But this difference was not observed following the secondary transplantation (Supplementary Figure 1E). There were no differences observed in the lineages of the spleen following primary transplantation or secondary transplantation, except in Mac1, which showed significant increase after secondary transplantation of KO BM cells (Supplementary Figures 1A and 1B). In tertiary transplant recipients, the differentiation towards B220 lineage was significantly reduced but there was an increased potential to differentiate into CD3 lineage (Supplementary Figure 1C). The lineage differentiation of BM cells in the transplant recipient mice of the primary and secondary transplant groups (Supplementary Figures 1D and 1E) showed a similar pattern as spleen, but the primary BM recipient had reduced B220 cells. However, tertiary recipient had reduced B220 lineage and increased Gr-1 positive cells along with a significant increase in the Mac1 lineage cells (Supplementary Figure 1F). In the BM of the primary transplant group (Figure 3(c)), we observed an increase in the phenotypic LTHSC population and a reduction in the early multipotent progenitor population (MPP2) of the secondary and tertiary transplant recipients. This suggests increased self-renewal potential of the AHR-depleted animals; this effect was more prominent in the secondary and tertiary transplant groups (Figures 3(d) and 3(e)). Together, these data suggest that absence of a functional AHR creates a hematopoietic stress-like condition and may simulate cues responsible for generating progenitor cells to reconstitute hematopoiesis.

### 3.4. Reciprocal Transplantation

**Figure 4:**
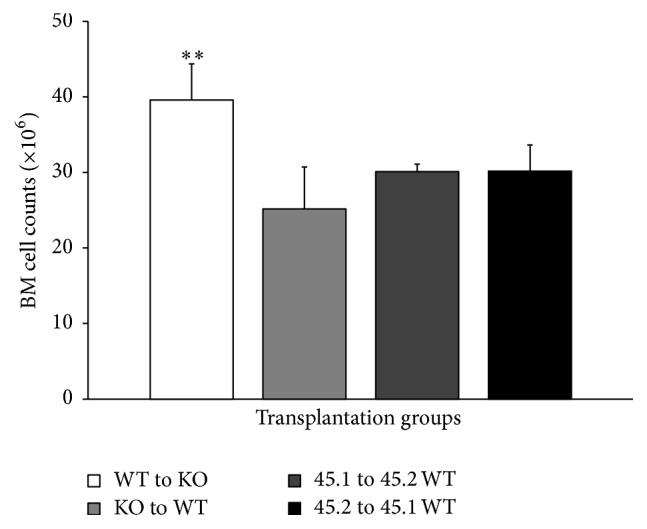
Lack of AHR expression in BM niche environment increases BM reconstitution ability of AHR expressing donor hematopoietic cells. Bone marrow chimera mice were prepared by lethal irradiation of WT (CD45.2^+^), WT (CD45.1^+^), and AHR-KO (CD45.2^+^) mice, as described in Section 2. Irradiated WT (CD45.1^+^) mice reconstituted with WT (CD45.2^+^) cells [WT (CD45.2^+^) → WT (CD45.1^+^)], irradiated WT (CD45.2^+^) mice reconstituted with WT (CD45.1^+^) cells [WT (CD45.1^+^) → WT (CD45.2^+^)], irradiated WT (CD45.1^+^) mice reconstituted with AHR-KO (CD45.2^+^) cells [AHR-KO (CD45.2^+^) → WT (CD45.1^+^)], and irradiated AHR-KO (CD45.2^+^) mice reconstituted with WT (CD45.1^+^) cells [WT (CD45.1^+^) → AHR-KO (CD45.2^+^)] are presented. Data shown are mean ± SD, *n* = 6–8 mice/group. ^*∗*^Values significantly different from WT control (*P* < 0.05).

The above data suggest that, consistent with previous investigations [14], deletion of AHR results in increased HSC proliferation. As such, we examined the possible involvement of cell extrinsic factors affecting HSC function in the absence of AHR by creating chimeric animals with and without AHR in hematopoietic and nonhematopoietic (including BM niche) cells. We transplanted WT cells into KO animals [18] and KO cells into WT animals as controls to analyze the BM repopulation. It is known that BM stromal cells contribute to the vascular niche in bone and are responsible for regulating the balance between self-renewal and differentiation of HSCs [19–21]. These stromal cells produce cytokines or extrinsic factors that influence hematopoietic self-renewal and differentiation. Following 16 weeks after transplant, there was a significant increase in BM cell counts only in the group that had donor WT (CD45.1) cells injected into recipient KO (CD45.2) animals (WT to KO group) (Figure 4). We did not observe any significant differences in the relative percentage of donor derived lineage specific populations (data not shown). These results indicate that the donor WT cells were able to repopulate more efficiently when the AHR is absent in the niche compared to other groups containing AHR in the niche. This suggests that BM niche factors provided by the KO microenvironment may be promoting the proliferation of HSCs.

### 3.5. Lack of AHR Alters the Cellular Composition of BM Stroma

**Figure 5:**
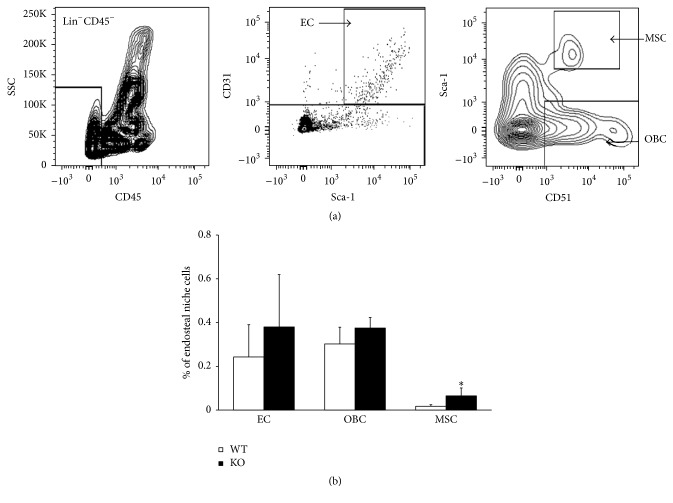
Lack of AHR expression alters the niche population. (a) Gating of nonhematopoietic endosteal niche cells. Endosteal niche cells are separated as Lin^−^CD45^−^, endothelial cells (EC) were gated as Lin^−^CD45^−^CD31^+^ cells, and MSC (mesenchymal stem cells) and OB (osteoblast) cells were gated from the Lin^−^CD45^−^CD31^−^ population based on Sca-1 and CD51 markers. The MSC are Lin^−^CD45^−^CD31^−^Sca-1^+^CD51^+^ and OB cells are Lin^−^CD45^−^CD31^−^Sca-1^−^CD51^+^. (b) represents the percentage of the endosteal niche cells from total endosteal BM stromal cells. Data are mean ± SD, *n* = 5 mice/group. ^*∗*^Values significantly different from WT control (*P* < 0.05).

The present and previous [14] data suggest that AHR regulates hematopoiesis by balancing, directly or indirectly, the quiescence and proliferation of HSCs. The fate of HSCs is influenced, in part, by signals emanating from endosteal niche cells [22]. We examined the relative presence of endosteal niche cells in both AHR-KO and WT animals. We gated cells from the lineage negative population, further selected for endothelial cells, and the remaining cells were gated for mesenchymal stromal cells and osteoblast cells (Figure 5(a)). There was a 3-fold increase (*P* = 0.003) in the number of mesenchymal stem cells in KO as compared to WT animals (Figure 5(b)). The endothelial and osteoblastic cells also showed a slight increase in numbers, but these differences were not statistically significant.

### 3.6. AHR Regulates the Expression of Genes Involved in HSC Maintenance

**Figure 6:**
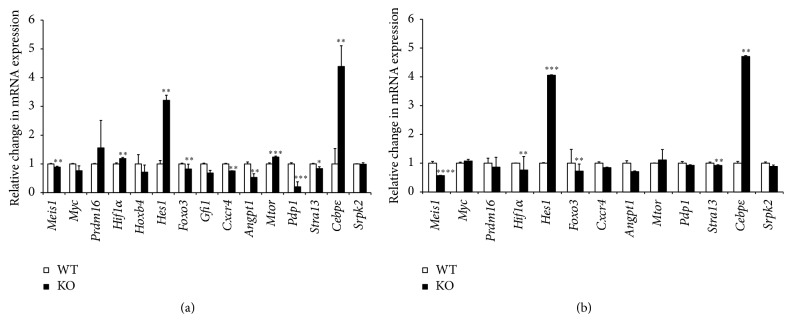
AHR-deficient LSK cells have alterations in gene expression related to hematopoiesis and hematopoietic disease. Quantitative real time PCR was used to analyze mRNA expression of genes associated with hematopoiesis and hematopoietic disease in LSK cells. Differential gene expression analysis of the LSK population in (a) young and (b) old mice. Data are mean ± SD, *n* = 5 mice/group. ^*∗*^Values significantly different from WT control (*P* < 0.05).

The LSK population from BM of WT and AHR-KO animals was sorted and the cDNA was subjected to gene expression analysis. This analysis focused on genes (*Srpk2*,* Hes1, Mtor, Pdp1, Meis1, Gfi1, Foxo3, Stra13, Hif1α, and Cebpε*) involved in HSC proliferation; maintenance of quiescence, growth, and stem cell exhaustion; and development of myeloproliferative disorder in young and geriatric mice. We also analyzed the expression of genes (*Cxcr4* and* Angpt1*) involved in regulating niche interactions. Notably, there was an increase in the expression of* Hes1* and* Cebpε* in both young and aged (Figures 6(a) and 6(b)) KO animals. The expression of genes (*Cxcr4* and* Angpt1*) involved in niche maintenance was significantly downregulated in young and old KO animals. The higher level of* Hes1* in both KO groups may reflect the preservation of long term reconstituting ability [16]. The level of* Meis1* was also downregulated in these groups which may be related to altered* HIf1α* and reduction in* Stra13* gene expression involved in balancing ROS production [23]. There was a significant increase in the expression of* Cebpε* in both the KO groups, suggesting involvement with mature granulocyte production [24].

## 4. Discussion

In this study, we assessed the role of AHR in hematopoiesis and initiated experiments to examine the possible effect of the BM niche microenvironment in regulating hematopoiesis in absence of AHR protein. Previous reports indicating a role of AHR in regulating immune system pathways, and hematopoiesis in particular, used mouse models in which exon 2 of the* Ahr* gene was deleted [25]. In the present report, we present data from AHR-KO mice generated by deletion of exon 3, resulting in an out of frame splicing of exons 2 to exon 4. In terms of the effects of AHR absence on hematopoiesis, this exon 3 deletion mouse model appears to be similar to the “Bradfield” AHR-KO mice evaluated in previous investigations (Table 1). Combined, these data indicate that AHR signaling has a significant role in hematopoiesis by mediating and/or balancing the proliferation and maintenance of HSCs and progenitor populations in BM. Here, we also present preliminary data implicating a role of AHR in regulating the niche population that influences the proliferation and differentiation of HSCs.

Previously, it was reported that AHR-KO mice have enlarged spleens and increased number of lineage positive spleen cells and altered number of white blood cells and red blood cells, as well as increased numbers and altered functions of BM progenitors/HSCs [14]. The BM HSCs from the KO animals were highly proliferative. HSCs from AHR-KO mice have increased expression of several chemokines, cytokines, and their receptor genes. HSCs from young AHR-KO have overexpression of* Srpk2, Creb1, Hes1, Mtor*, and* Pdp1*. These genes have been associated with oxidative stress, acute myelogenous leukemia, aging and heat shock response, and alterations in the *β*-catenin/Wnt pathways. Earlier reports from AHR-KO mice indicated changes in multiple signaling pathways that promote premature HSC exhaustion and development of myeloproliferative disorder. Aging AHR-KO mice have changes in several aging associated genes that may result in shorter lifespan [12, 13].

Our results from the exon 3 deletion model show increased weights of spleen and decreased weights of liver in AHR-KO mice, as well as increased proliferation of HSCs (Figures 1(d) and 1(e)). The aging mice showed significant increase in monocyte and granulocyte numbers in peripheral blood (Figures 1(a) and 1(b)). This change may reflect skewing of lineage towards myeloid differentiation as opposed to lymphoid, reflecting early sign of aging as observed in earlier reports [13].

Consistent with previous studies [14], we found that AHR-KO mice have significant increase in numbers of LSK cells. The BrdU incorporation data and the cell cycle data showing reduced cell numbers in G0 phase indicated hyperproliferation in the AHR-KO BM progenitor and/or stem cells. Similar results were observed following the treatment of human hematopoietic progenitor/stem cells with AHR antagonists [26]. Further studies are needed to delineate the mechanisms by which AHR appears to influence the balance between quiescence and proliferation.

The repopulation potential of HSCs depends upon self-renewal, differentiation, and homing abilities. To better understand these HSC functions, we performed long term serial bone marrow transplantation using BM cells from AHR-KO and WT mice. These studies support a significant role of AHR in the maintenance of HSC self-renewal and quiescence. These results also indicate that a most primitive LTHSC population needs AHR for its functional stability and in the absence of AHR a condition resembling hematopoietic stress is created triggering the cells to mobilize and proliferate. The increase in LTHSC in KO transplant recipient animals correlates with the reduction in MPP2 populations and this could be due to a differentiation block in these LTHSC populations. This might account for the increased proliferation and exhaustion of LSK cells in KO animals leading to poor survival compared to WT animals. We also found a reduced survival rate (66%) in the 10-month-old KO animals compared to WT littermates (data not shown), supporting an earlier observation [13]. There are also reports suggesting increased HSC proliferation rates in AHR-KO mice that are also prone to early senescence and decreased lifespan [11].

The increased proliferation of cells in KO animals might indicate that altered signals emanating from the BM niche might influence cell proliferation and maintenance of quiescence in LTHSC. It is known that the BM microenvironment (the niche) regulates the quiescence, proliferation, and differentiation of HSCs [3]. Consistent with this, our preliminary studies show that loss of AHR has an impact on the niche population as indicated by the changes in the heterogeneous niche cells. The osteoblast is known to regulate the niche [15]. Endothelial cells support hematopoiesis through expression of several molecules including Angiopoietin 1 (Angpt1). This is also expressed by osteoblasts and promotes the maintenance of quiescent HSCs in osteoblastic niche [27]. It has been recently reported that* Angpt1* is highly expressed by HSCs, facilitating regeneration of BM niche by secreting Angpt1. Notably, we observed a reduction in the* Angpt1* gene expression in LSK cells in KO animals (Figure 6). Thus, AHR absence in niche cells may be affecting the secretion of these factors supporting hematopoiesis. It has been reported that change in HSCs may induce secondary changes in BM niche cells function [28]. The observation of higher BM cell counts in chimeric mice having WT hematopoietic cells transplanted into AHR-KO hosts (Figure 4) is further evidence that lack of AHR within the BM stroma may also have a role in the HSC phenotype of the AHR-KO mice.

We performed chimeric experiments using AHR positive (WT) and negative (AHR-KO) transplantation recipient mice to further support a likely role of AHR in BM niche cells in hematopoiesis. These results provided additional evidence for the role of AHR in hematopoiesis through microenvironment signals operating through osteoblast and mesenchymal stem cells. Presently, mechanisms responsible for the delicate balance of BM cells to differentiate and proliferate rapidly are uncertain. However, the role of secretory products and direct contact signaling from the niche cells may be partly responsible for these changes. Future studies are needed to delineate the role of secretory products in the niche environment and the signaling mediated by AHR in the internal milieu of the niche cells contributing to hematopoiesis at steady state and under stress conditions.

Additional gene expression analysis of LSK population indicates changes in* Mtor*,* Hes1, PdP-1,* and* Stra13* related to various aspects of HSC maintenance. Upregulation of* mTOR* expression is associated with accelerated aging processes and aging associated diseases [29]. We observed changes in* Pdp-1* expression which acts as negative modulator of insulin/IGF-1 pathway (IIS). The IIS pathway is a regulator of longevity, development, and metabolism. It has been reported that Pdp1 may mediate this function in part by negatively regulating TGF-*β* signaling to repress expression of several insulins that feed into the IIS pathway. Dysregulation of TGF-*β* signaling and the IIS axis have been implicated in the onset of age-associated disease such as type 2 diabetes and cancer [30]. There was a significant increase in Hes1 in KO animals, suggesting its role in preserving the quiescence of LTHSCs [16]. It has been proposed that Hes1 is a positive regulator for the expansion of HSCs without exhausting their stem cell activity [31]. Stra13 is involved in regulating the oxidative stress in skeletal muscles [23] and we have seen increased ROS production in these animals in our previous publications.

Our studies also indicated a significant upregulation of* Cebpε* expression that may be related to altered granulocyte production in the KO animals [24]. We also observed an alteration in* Hif1α* mRNA levels in KO animals that is likely to influence the proliferation of HSCs [32]. We have also noted downregulation of* Meis1* expression involved in regulating the oxidative stress [33]. AHR mice have shown signs of early aging and myeloproliferative disease [13]. Aging AHR-KO mice have impaired glucose and lipid metabolism [34]. The most significant upregulation was observed in* Hes1* and* Cebpε* genes in both young and aging AHR-KO mice; these alterations may be associated with HSCs quiescence and granulocyte production [16, 24].

## 5. Conclusion

In conclusion, here we present data from AHR-KO mice generated by deletion of exon 3 in the AHR gene. In terms of hematopoietic effects, this mouse model of AHR-KO appears to be similar to “Bradfield” AHR-KO mice as evaluated by the parameters reported here. Taken together, our results indicate that AHR has a definitive role in the regulation of HSCs functions. Altered AHR signaling appears to produce significant effects on hematopoiesis by modulating the proliferation and maintenance of HSCs and progenitor populations in the blood and BM. The observed changes in functions of AHR-deficient HSCs may be due, in part, to changes in the AHR-KO niche cells from where critical regulatory signals emanate.

## Supplementary Material

AHR also regulates HSC differentiation in transplantation recipient animals. We performed serial transplantation assay to assess the role of AHR in differentiation and homing of HSCs. We analyzed the different lineages in the spleen and bone marrow of the transplantation recipient animals after 16 weeks post transplantation. We found a significant increase in the CD3 and decrease in B220 lineages of the AHR KO spleens of tertiary recipients. The bone marrow showed decrease in B220 and increase in Gr1 and Mac1 lineages. These changes in BM reflect myeloid-biased differentiation which is classical sign of aging. The myeloid-biased HSCs have extensive self-renewal capacity but diminished ability to differentiate to lymphoid cells (Supplementary Figure 1). Histological examination of spleen showed increased cellularity and lymphoid follicle number in AHR KO mice. Increased thickness of cortical area in thymus and portal fibrosis in livers of AHR KO mice was also observed (Supplementary Figure 2).

## Figures and Tables

**Table 1:** AHR influences HSC characteristics.

Hematopoietic characteristics	AHR exon 2 KO mice (Bradfield) [14]	AHR exon 3 KO mice (Taconic Biosciences)
Platelet counts	NR	Increased
WBC count	Increased	Higher but NS
Granulocyte and monocyte population	Reduced	Increased but NS
RBC number	Reduced	No change
Liver weight	Reduced	Reduced
Spleen weight and cell number	Increased	Increased
Functional primitive progenitor colonies	Increased numbers	Increased numbers
LSK cells	Increased	Increased
LSK cell proliferation	Higher	Higher
Cell cycle status of LSK cells	Reduced G0/G1 phase Increased G2/M phase	Reduced G0/G1 phase Increased G2/M phase
LTHSC in BM	NR	Higher
Increased self-renewal of LTHSC (serial transplantation)	ND	Higher
Mesenchymal stem cells in endosteal niche	ND	Higher
Survival rate	Reduced	Reduced
Gene expression changes	*Mtor* (↑) *Cebpε* (↑) *Gfi1* (↓) *Hes1* (↑) *Stra13* (↓)	*Mtor* (↑) *Cebpε* (↑) *Gfi1* (↓) *Hes1* (↑) *Stra13* (↓)

The table depicts the difference and similarities between the two different strains of AHR-KO mice models (NR = not recorded, ND = not done, NS = not significant, (↑) = upregulation, and (↓) = downregulation).
