# Supplementary figures and images for: Aryl Hydrocarbon Receptor Deficiency in an Exon 3 Deletion Mouse Model Promotes Hematopoietic Stem Cell Proliferation and Impacts Endosteal Niche Cells
Source: Stem Cells Int. 2016 Jun 16;2016:4536187. doi: 10.1155/2016/4536187 (PMC4913018; doi:10.1155/2016/4536187)

## Slide 1
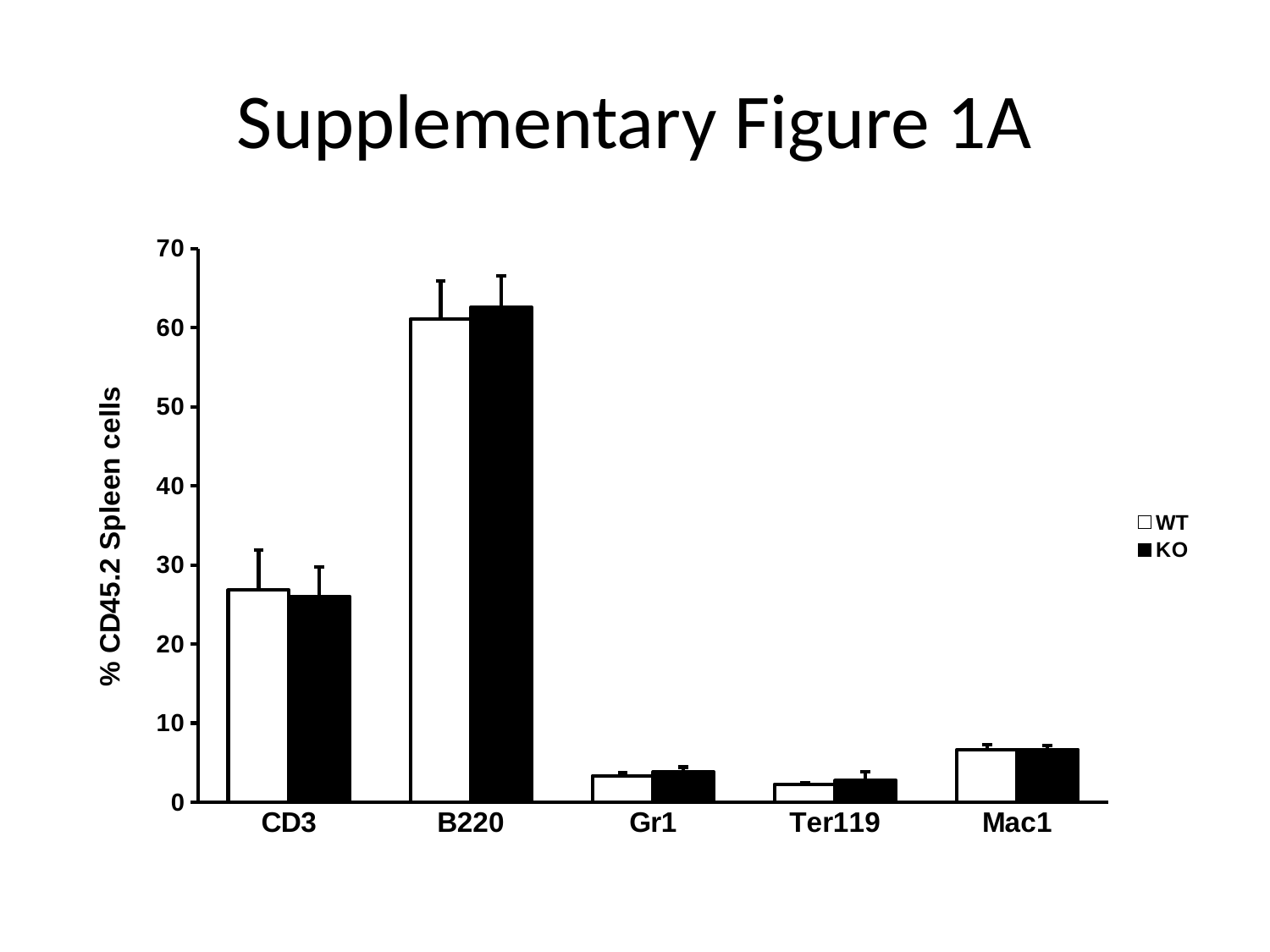

# Supplementary Figure 1A
### Chart
| Category | | |
|---|---|---|
| CD3 | 26.837500000000002 | 26.03333333333333 |
| B220 | 61.0875 | 62.583333333333336 |
| Gr1 | 3.3600000000000003 | 3.8433333333333333 |
| Ter119 | 2.28875 | 2.7916666666666665 |
| Mac1 | 6.61375 | 6.6816666666666675 |

Supplement: Supplementary file 1 — AHR also regulates HSC differentiation in transplantation recipient animals. We performed serial transplantation assay to assess the role of AHR in differentiation and homing of HSCs. We analyzed the different lineages in the spleen and bone marrow of the transplantation recipient animals after 16 weeks post transplantation. We found a significant increase in the CD3 and decrease in B220 lineages of the AHR KO spleens of tertiary recipients. The bone marrow showed decrease in B220 and increase in Gr1 and Mac1 lineages. These changes in BM reflect myeloid-biased differentiation which is classical sign of aging. The myeloid-biased HSCs have extensive self-renewal capacity but diminished ability to differentiate to lymphoid cells (Supplementary Figure 1). Histological examination of spleen showed increased cellularity and lymphoid follicle number in AHR KO mice. Increased thickness of cortical area in thymus and portal fibrosis in livers of AHR KO mice was also observed (Supplementary Figure 2). [file 4536187.f1.zip › Supplementary_Figure_1A_SCI_1657161.pptx]

## Slide 1
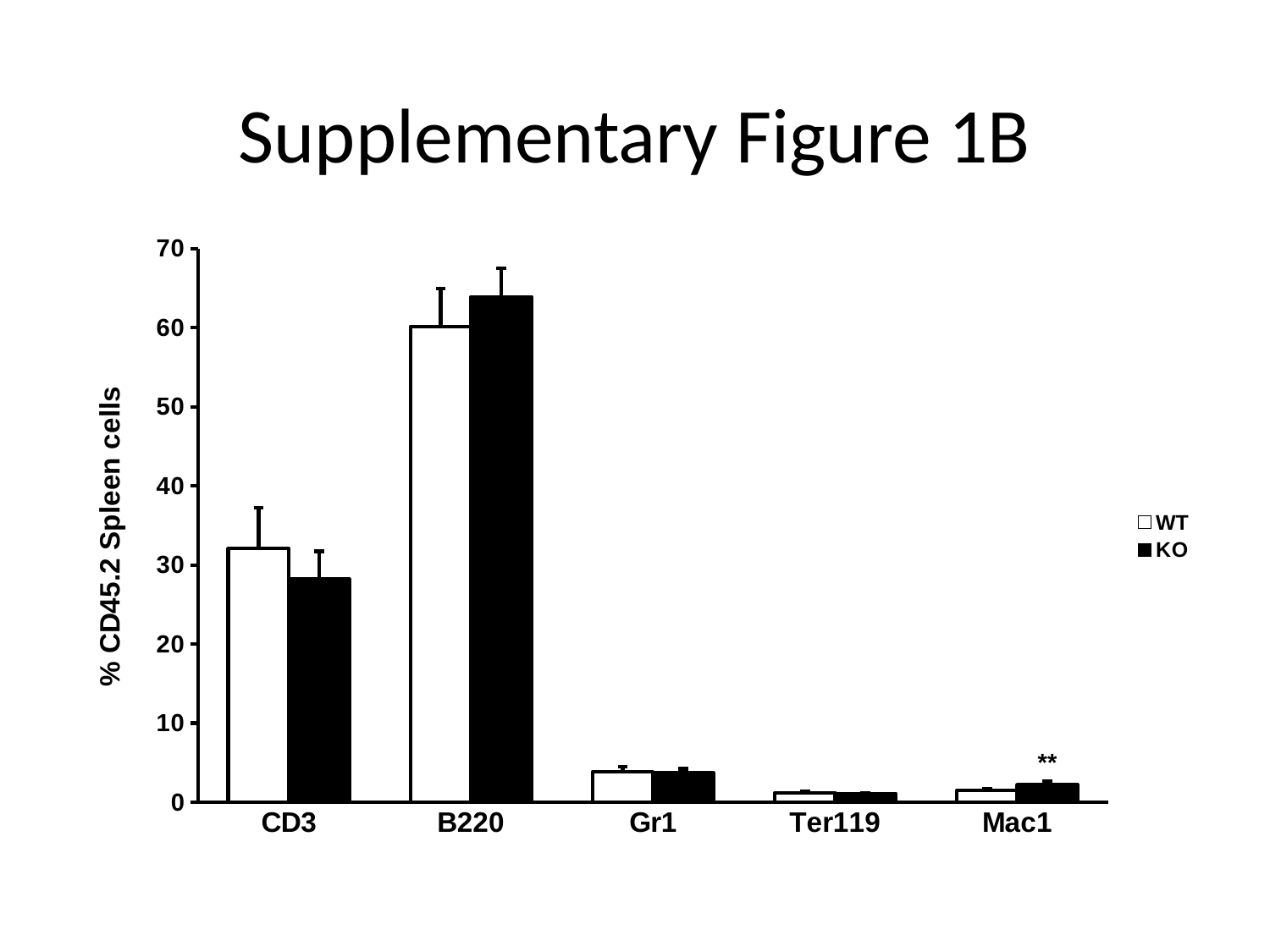

# Supplementary Figure 1B
### Chart
| Category | | |
|---|---|---|
| CD3 | 32.06666666666667 | 28.199999999999996 |
| B220 | 60.166666666666664 | 63.91428571428572 |
| Gr1 | 3.842 | 3.6900000000000004 |
| Ter119 | 1.1816666666666666 | 1.1014285714285714 |
| Mac1 | 1.4799999999999998 | 2.2028571428571433 |**

Supplement: Supplementary file 1 — AHR also regulates HSC differentiation in transplantation recipient animals. We performed serial transplantation assay to assess the role of AHR in differentiation and homing of HSCs. We analyzed the different lineages in the spleen and bone marrow of the transplantation recipient animals after 16 weeks post transplantation. We found a significant increase in the CD3 and decrease in B220 lineages of the AHR KO spleens of tertiary recipients. The bone marrow showed decrease in B220 and increase in Gr1 and Mac1 lineages. These changes in BM reflect myeloid-biased differentiation which is classical sign of aging. The myeloid-biased HSCs have extensive self-renewal capacity but diminished ability to differentiate to lymphoid cells (Supplementary Figure 1). Histological examination of spleen showed increased cellularity and lymphoid follicle number in AHR KO mice. Increased thickness of cortical area in thymus and portal fibrosis in livers of AHR KO mice was also observed (Supplementary Figure 2). [file 4536187.f1.zip › Supplementary_Figure_1B_SCI_1657162.pptx]

## Slide 1
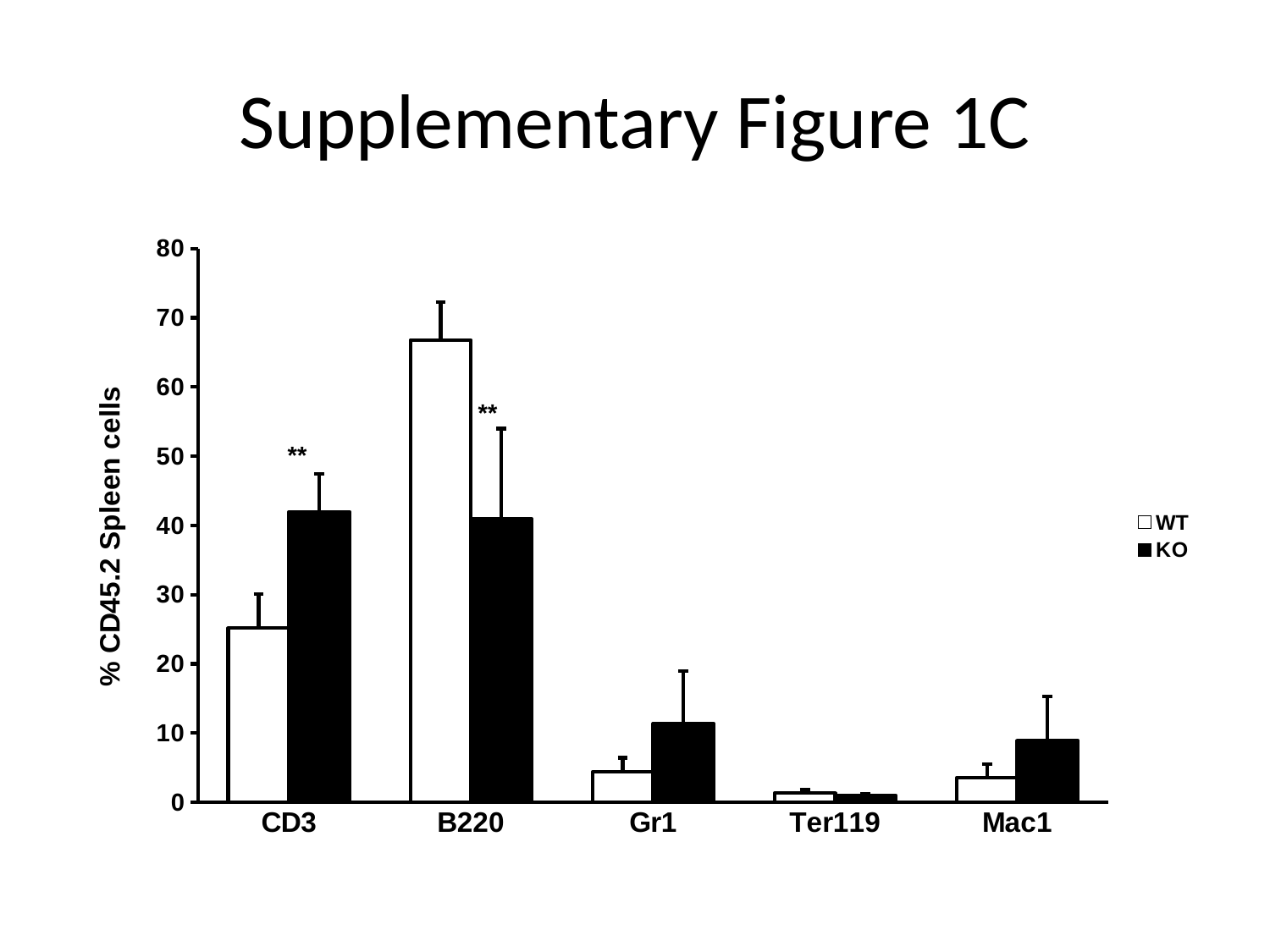

# Supplementary Figure 1C
### Chart
| Category | | |
|---|---|---|
| CD3 | 25.183333333333334 | 41.96666666666667 |
| B220 | 66.78333333333335 | 40.93333333333333 |
| Gr1 | 4.386666666666667 | 11.37 |
| Ter119 | 1.3083333333333333 | 0.9833333333333333 |
| Mac1 | 3.52 | 8.893333333333333 |**
**

Supplement: Supplementary file 1 — AHR also regulates HSC differentiation in transplantation recipient animals. We performed serial transplantation assay to assess the role of AHR in differentiation and homing of HSCs. We analyzed the different lineages in the spleen and bone marrow of the transplantation recipient animals after 16 weeks post transplantation. We found a significant increase in the CD3 and decrease in B220 lineages of the AHR KO spleens of tertiary recipients. The bone marrow showed decrease in B220 and increase in Gr1 and Mac1 lineages. These changes in BM reflect myeloid-biased differentiation which is classical sign of aging. The myeloid-biased HSCs have extensive self-renewal capacity but diminished ability to differentiate to lymphoid cells (Supplementary Figure 1). Histological examination of spleen showed increased cellularity and lymphoid follicle number in AHR KO mice. Increased thickness of cortical area in thymus and portal fibrosis in livers of AHR KO mice was also observed (Supplementary Figure 2). [file 4536187.f1.zip › Supplementary_Figure_1C_SCI_1657163.pptx]

## Slide 1
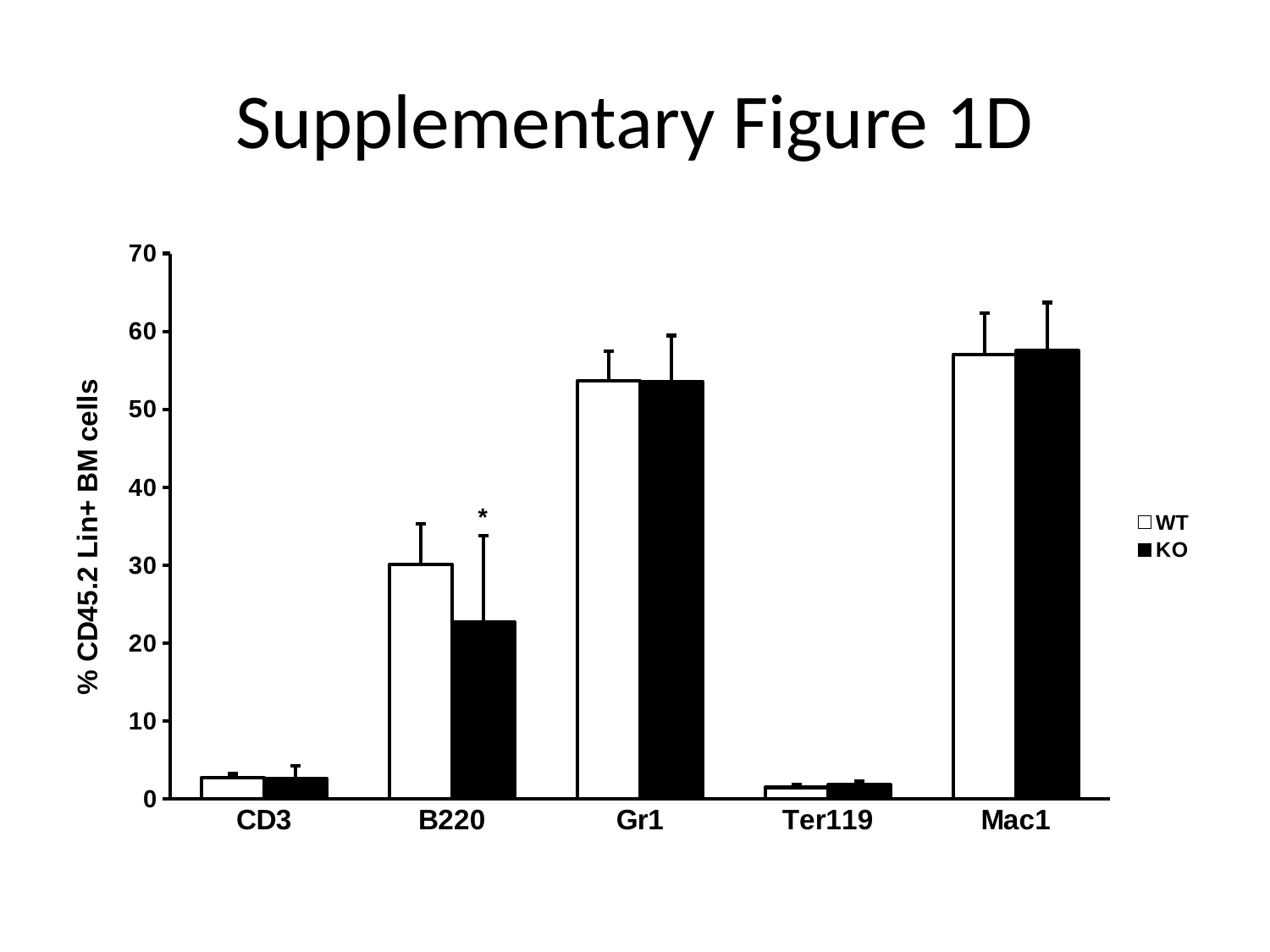

# Supplementary Figure 1D
### Chart
| Category | | |
|---|---|---|
| CD3 | 2.74 | 2.6042857142857136 |
| B220 | 30.062500000000004 | 22.685714285714287 |
| Gr1 | 53.6375 | 53.528571428571425 |
| Ter119 | 1.4975 | 1.8228571428571425 |
| Mac1 | 57.02499999999999 | 57.51428571428571 |

Supplement: Supplementary file 1 — AHR also regulates HSC differentiation in transplantation recipient animals. We performed serial transplantation assay to assess the role of AHR in differentiation and homing of HSCs. We analyzed the different lineages in the spleen and bone marrow of the transplantation recipient animals after 16 weeks post transplantation. We found a significant increase in the CD3 and decrease in B220 lineages of the AHR KO spleens of tertiary recipients. The bone marrow showed decrease in B220 and increase in Gr1 and Mac1 lineages. These changes in BM reflect myeloid-biased differentiation which is classical sign of aging. The myeloid-biased HSCs have extensive self-renewal capacity but diminished ability to differentiate to lymphoid cells (Supplementary Figure 1). Histological examination of spleen showed increased cellularity and lymphoid follicle number in AHR KO mice. Increased thickness of cortical area in thymus and portal fibrosis in livers of AHR KO mice was also observed (Supplementary Figure 2). [file 4536187.f1.zip › Supplementary_Figure_1D_SCI_1657164.pptx]

## Slide 1
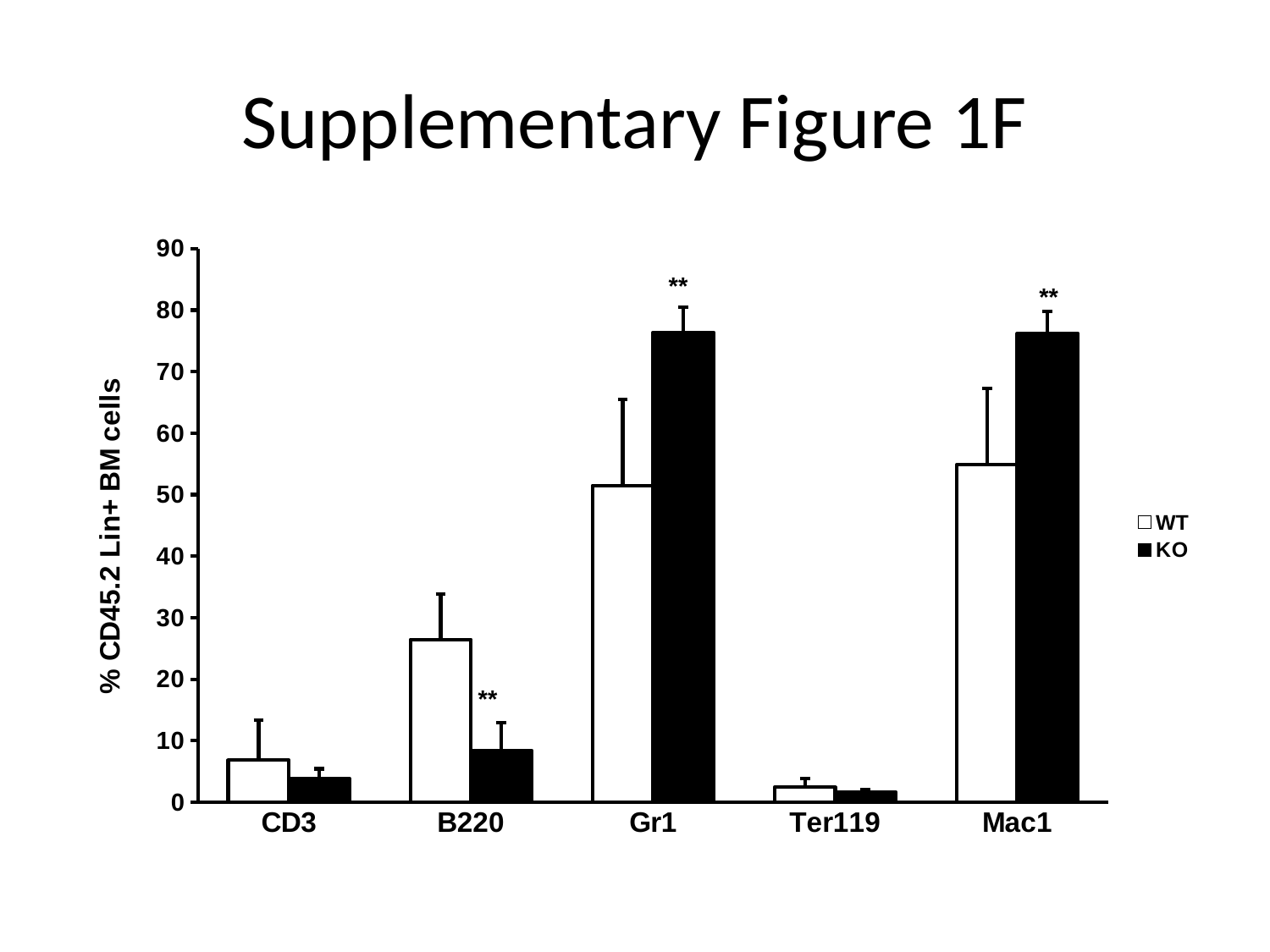

# Supplementary Figure 1F
### Chart
| Category | | |
|---|---|---|
| CD3 | 6.847999999999999 | 3.854 |
| B220 | 26.450000000000003 | 8.41 |
| Gr1 | 51.5 | 76.38 |
| Ter119 | 2.47 | 1.682 |
| Mac1 | 54.86 | 76.22 |**
**
**

Supplement: Supplementary file 1 — AHR also regulates HSC differentiation in transplantation recipient animals. We performed serial transplantation assay to assess the role of AHR in differentiation and homing of HSCs. We analyzed the different lineages in the spleen and bone marrow of the transplantation recipient animals after 16 weeks post transplantation. We found a significant increase in the CD3 and decrease in B220 lineages of the AHR KO spleens of tertiary recipients. The bone marrow showed decrease in B220 and increase in Gr1 and Mac1 lineages. These changes in BM reflect myeloid-biased differentiation which is classical sign of aging. The myeloid-biased HSCs have extensive self-renewal capacity but diminished ability to differentiate to lymphoid cells (Supplementary Figure 1). Histological examination of spleen showed increased cellularity and lymphoid follicle number in AHR KO mice. Increased thickness of cortical area in thymus and portal fibrosis in livers of AHR KO mice was also observed (Supplementary Figure 2). [file 4536187.f1.zip › Supplementary_Figure_1F_SCI_1657165.pptx]

## Slide 1
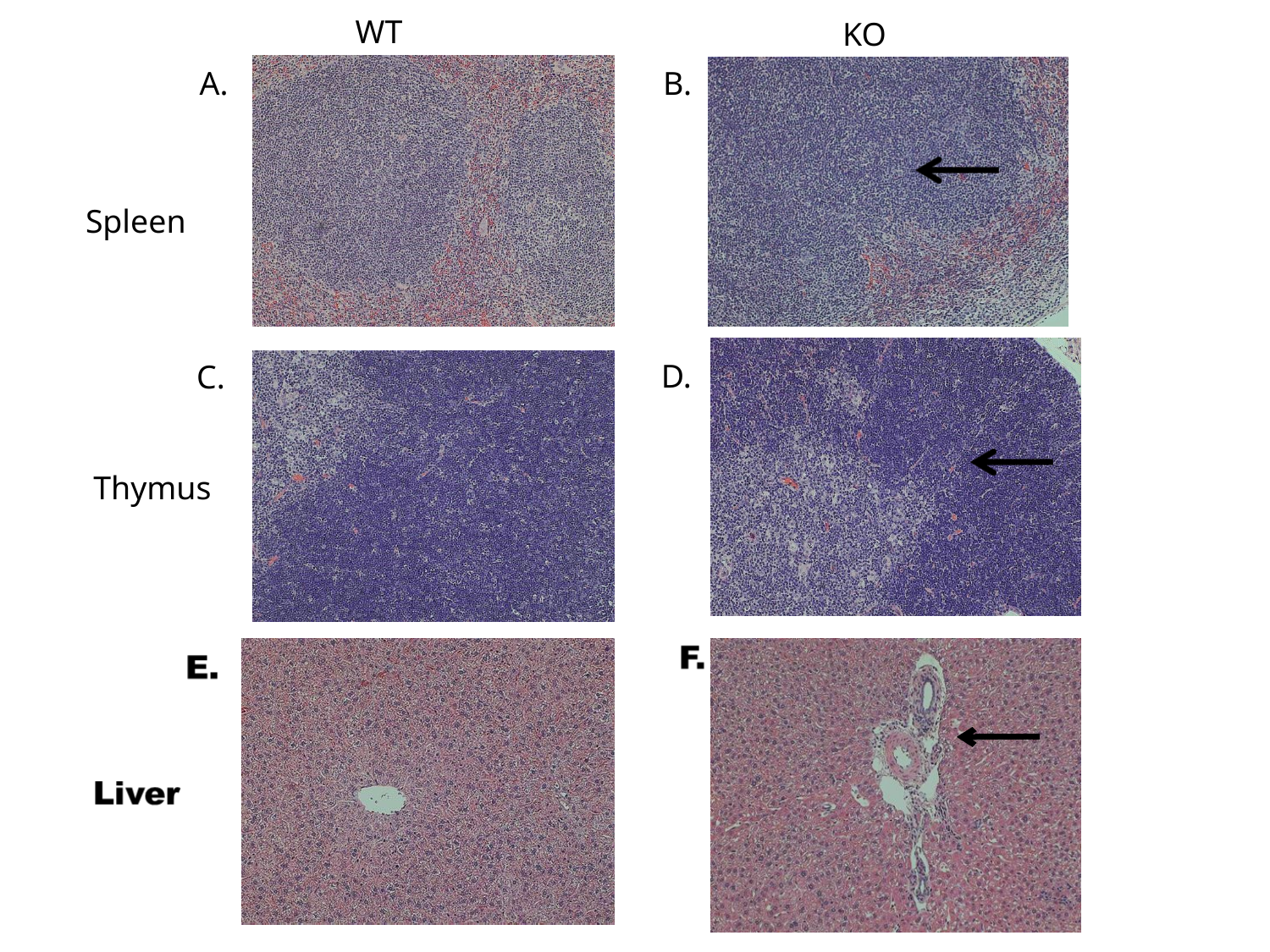

WT
KO
A.
B.
Spleen
D.
C.
Thymus

Supplement: Supplementary file 1 — AHR also regulates HSC differentiation in transplantation recipient animals. We performed serial transplantation assay to assess the role of AHR in differentiation and homing of HSCs. We analyzed the different lineages in the spleen and bone marrow of the transplantation recipient animals after 16 weeks post transplantation. We found a significant increase in the CD3 and decrease in B220 lineages of the AHR KO spleens of tertiary recipients. The bone marrow showed decrease in B220 and increase in Gr1 and Mac1 lineages. These changes in BM reflect myeloid-biased differentiation which is classical sign of aging. The myeloid-biased HSCs have extensive self-renewal capacity but diminished ability to differentiate to lymphoid cells (Supplementary Figure 1). Histological examination of spleen showed increased cellularity and lymphoid follicle number in AHR KO mice. Increased thickness of cortical area in thymus and portal fibrosis in livers of AHR KO mice was also observed (Supplementary Figure 2). [file 4536187.f1.zip › Supplementary_Figure_2_SCI_1657166.pptx]

## Slide 1
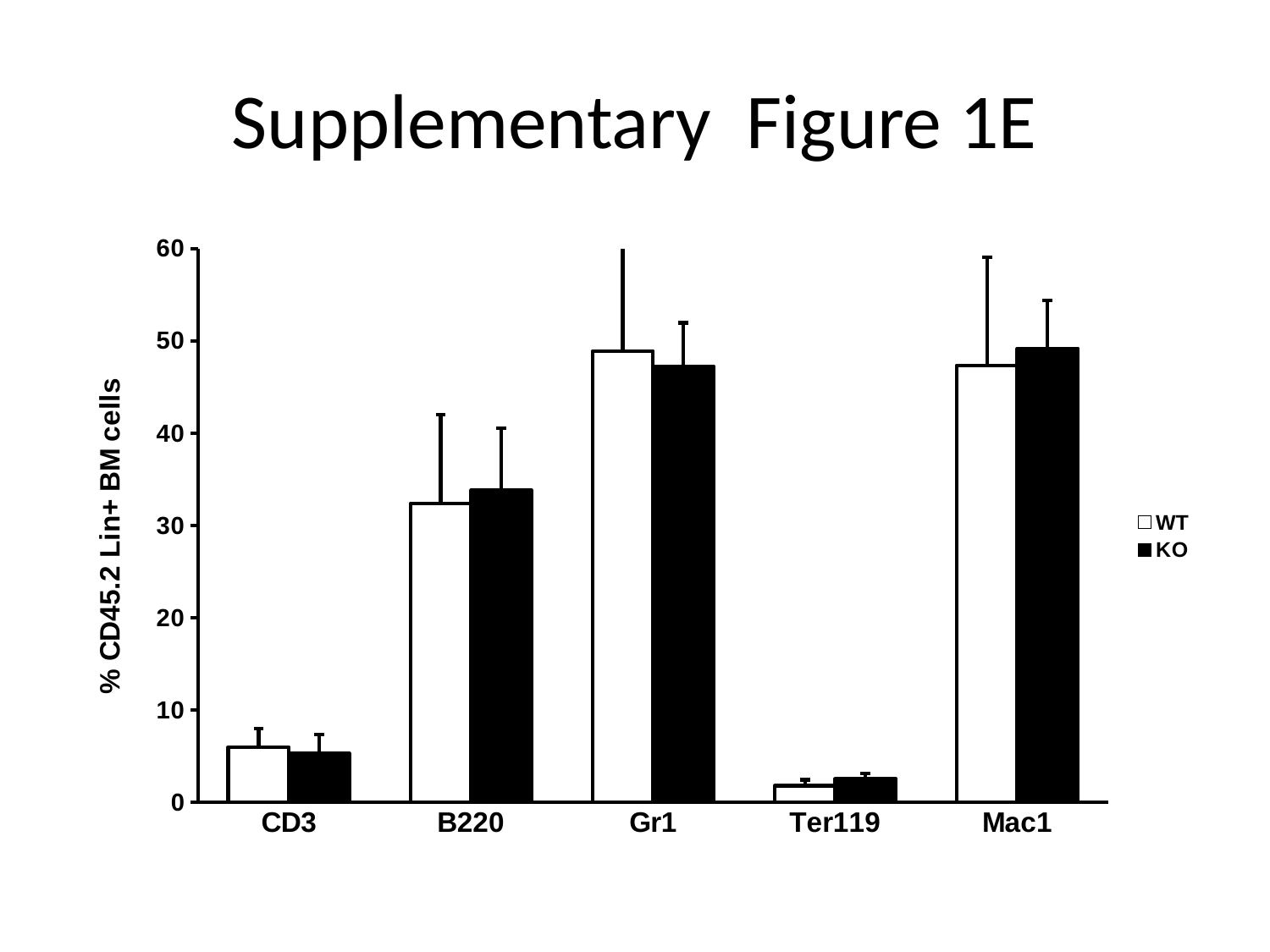

# Supplementary Figure 1E
### Chart
| Category | | |
|---|---|---|
| CD3 | 5.985 | 5.284285714285716 |
| B220 | 32.4 | 33.800000000000004 |
| Gr1 | 48.9 | 47.24285714285714 |
| Ter119 | 1.8028571428571427 | 2.54 |
| Mac1 | 47.357142857142854 | 49.18571428571429 |

Supplement: Supplementary file 1 — AHR also regulates HSC differentiation in transplantation recipient animals. We performed serial transplantation assay to assess the role of AHR in differentiation and homing of HSCs. We analyzed the different lineages in the spleen and bone marrow of the transplantation recipient animals after 16 weeks post transplantation. We found a significant increase in the CD3 and decrease in B220 lineages of the AHR KO spleens of tertiary recipients. The bone marrow showed decrease in B220 and increase in Gr1 and Mac1 lineages. These changes in BM reflect myeloid-biased differentiation which is classical sign of aging. The myeloid-biased HSCs have extensive self-renewal capacity but diminished ability to differentiate to lymphoid cells (Supplementary Figure 1). Histological examination of spleen showed increased cellularity and lymphoid follicle number in AHR KO mice. Increased thickness of cortical area in thymus and portal fibrosis in livers of AHR KO mice was also observed (Supplementary Figure 2). [file 4536187.f1.zip › Supplementary__Figure_1E_SCI_1657160.pptx]
